# Supplementary material for: WDR90 is a centriolar microtubule wall protein important for centriole architecture integrity
Source: eLife. 2020 Sep 18;9:e57205. doi: 10.7554/eLife.57205 (PMC7500955; doi:10.7554/eLife.57205)
Supplement: Figure 3—source data 1. [file elife-57205-fig3-data1.docx]

|  | **Time** | | |
| --- | --- | --- | --- |
| **Percentage of cells** | **14hrs** | **22hrs** | **24hrs** |
| **2--0** | 57 +/- 2 | 20 +/- 1 | 10 +/- 3 |
| **2--2** | 38 +/- 4 | 59 +/- 1 | 57 +/- 4 |
| **4--2** | 5 +/- 13 | 21 +/- 15 | 33 +/- 45 |

**Figure 3-source data 1:** Percentage of cells with the following number of dots/cell respectively for WDR90 and Centrin.
