# Supplementary material for: WDR90 is a centriolar microtubule wall protein important for centriole architecture integrity
Source: eLife. 2020 Sep 18;9:e57205. doi: 10.7554/eLife.57205 (PMC7500955; doi:10.7554/eLife.57205)
Supplement: Figure 3—source data 2. [file elife-57205-fig3-data2.docx]

|  | **Time** | | |
| --- | --- | --- | --- |
| **Percentage of cells** | **14hrs** | **22hrs** | **24hrs** |
| **2--0** | 53 +/-3 | 19 +/- 6 | 9 +/- 3 |
| **2--2** | 44 +/- 4 | 67 +/- 1 | 67 +/- 3 |
| **4--2** | 3 +/- 1 | 13 +/- 1 | 24 +/- 4 |

**Figure 3-source data 2:** Percentage of cells with the following number of dots/cell respectively for WDR90 and HsSAS-6.
