# Supplementary material for: WDR90 is a centriolar microtubule wall protein important for centriole architecture integrity
Source: eLife. 2020 Sep 18;9:e57205. doi: 10.7554/eLife.57205 (PMC7500955; doi:10.7554/eLife.57205)
Supplement: Figure 3—figure supplement 1—source data 1. [file elife-57205-fig3-figsupp1-data1.docx]

|  | **Time** | | |
| --- | --- | --- | --- |
| **Percentage of cells** | **14hrs** | **22hrs** | **24hrs** |
| **G0/G1** | 47 +/- 10 | 19 +/- 10 | 14 +/- 5 |
| **S** | 43 +/- 4 | 39 +/- 13 | 17 +/- 5 |
| **G2/M** | 10 +/- 1 | 42 +/- 7 | 69 +/- 5 |

**Figure 3-figure supplement 1-source data 1:** Percentage of cells in each phase of the cell cycle according to post-mitotic time point
