# Supplementary material for: WDR90 is a centriolar microtubule wall protein important for centriole architecture integrity
Source: eLife. 2020 Sep 18;9:e57205. doi: 10.7554/eLife.57205 (PMC7500955; doi:10.7554/eLife.57205)
Supplement: Figure 3—figure supplement 2—source data 1. [file elife-57205-fig3-figsupp2-data1.docx]

| **% of cells** | **2 Centrin dots** | | | **4 Centrin dots** | | |
| --- | --- | --- | --- | --- | --- | --- |
|  | 0 WDR90 | 1 WDR90 | 2 WDR90 | 1 WDR90 | 2 WDR90 | 4 WDR90 |
| **siControl** | 0 +/- 0 | 10 +/- 2 | 90 +/- 2 | 0 +/- 0 | 71 +/- 1 | 29 +/- 1 |
| **siWDR90** | 0 +/- 0 | 69 +/- 7 | 31 +/- 7 | 77 +/- 5 | 23 +/- 5 | 0 +/- 0 |

**Figure 3-figure supplement 2-source data 1:** Percentage of cells displaying 0, 1, 2 or 4 dots of WDR90 based on the number of Centrin dots in U2OS cells treated with control or *wdr90* siRNA.
