# Supplementary material for: WDR90 is a centriolar microtubule wall protein important for centriole architecture integrity
Source: eLife. 2020 Sep 18;9:e57205. doi: 10.7554/eLife.57205 (PMC7500955; doi:10.7554/eLife.57205)
Supplement: Figure 3—figure supplement 2—source data 2. [file elife-57205-fig3-figsupp2-data2.docx]

| **% of cells** | **0 HsSAS-6 dot** | | | **2 HsSas-6 dots** | | |
| --- | --- | --- | --- | --- | --- | --- |
|  | 0 POC5 | 1 POC5 | 2 POC5 | 1 POC5 | 2 POC5 | 4 POC5 |
| **siControl** | 0 +/- 0 | 14 +/- 5 | 86 +/- 5 | 4 +/- 4 | 68 +/- 6 | 28 +/- 4 |
| **siWDR90** | 0 +/- 0 | 61 +/- 10 | 39 +/- 10 | 63 +/- 9 | 29 +/- 11 | 8 +/- 3 |

**Figure 3-figure supplement 2-source data 2:** Percentage of cells displaying 0, 1, 2 or 4 dots of POC5 based on the number of HsSas-6 dots in U2OS cells treated with control or *wdr90* siRNA.
