# Supplementary material for: WDR90 is a centriolar microtubule wall protein important for centriole architecture integrity
Source: eLife. 2020 Sep 18;9:e57205. doi: 10.7554/eLife.57205 (PMC7500955; doi:10.7554/eLife.57205)
Supplement: Figure 4—source data 1. [file elife-57205-fig4-data1.docx]

| **Diameter (nm)** | **siControl** | **siWDR90** |
| --- | --- | --- |
| **Proximal** | 243 +/-23 | 231 +/- 16 |
| **Core** | 244 +/-25 | 223 +/- 15 |
| **Distal** | 214 +/- 25 | 203 +/- 15 |

**Figure 4-source data 1:** Diameter at proximal, core and distal region of the centriole
