# Supplementary material for: WDR90 is a centriolar microtubule wall protein important for centriole architecture integrity
Source: eLife. 2020 Sep 18;9:e57205. doi: 10.7554/eLife.57205 (PMC7500955; doi:10.7554/eLife.57205)
Supplement: Figure 4—source data 2. [file elife-57205-fig4-data2.docx]

| **Coverage (%)** | **siControl** | **siWDR90** |
| --- | --- | --- |
| **POC1B** | 59 +/-14 | 34 +/- 16 |
| **FAM161A** | 61 +/- 9 | 18 +/- 7 |
| **POC5** | 56 +/- 11 | 17 +/- 9 |
| **Centrin** | 50 +/- 16 | 27 +/- 14 |

**Figure 4-source data 2:** Inner scaffold proteins coverage
