# Supplementary material for: WDR90 is a centriolar microtubule wall protein important for centriole architecture integrity
Source: eLife. 2020 Sep 18;9:e57205. doi: 10.7554/eLife.57205 (PMC7500955; doi:10.7554/eLife.57205)
Supplement: Figure 6—source data 1. [file elife-57205-fig6-data1.docx]

| **% of cells** | **Conditions** | |
| --- | --- | --- |
|  | **< 2 POC5 dots** | **≥ 2 POC5 dots** |
| **siControl** | **1.1 +/- 1.9** | **98.9 +/- 1.9** |
| **siPOC5** | **82 +/- 7** | **18 +/- 7** |

**Figure 6-source data 1:** Percentage of cells with the following number POC5 dots/cell in siControl and siPOC5 conditions.
