# Supplementary material for: WDR90 is a centriolar microtubule wall protein important for centriole architecture integrity
Source: eLife. 2020 Sep 18;9:e57205. doi: 10.7554/eLife.57205 (PMC7500955; doi:10.7554/eLife.57205)
Supplement: Figure 6—source data 2. [file elife-57205-fig6-data2.docx]

| **% of cells** | **Conditions** | |
| --- | --- | --- |
|  | **< 2 WDR90 dots** | **≥ 2 WDR90 dots** |
| **siControl** | **1 +/- 1.4** | **99 +/- 1.4** |
| **siPOC5** | **3 +/- 1.4** | **97 +/- 1.4** |

**Figure 6-source data 2:** Percentage of cells with the following number WDR90 dots/cell in siControl and siPOC5 conditions.
