# Supplementary material for: WDR90 is a centriolar microtubule wall protein important for centriole architecture integrity
Source: eLife. 2020 Sep 18;9:e57205. doi: 10.7554/eLife.57205 (PMC7500955; doi:10.7554/eLife.57205)
Supplement: Figure 6—figure supplement 1—source data 1. [file elife-57205-fig6-figsupp1-data1.docx]

| **Tubulin length (nm)** | **Conditions** | |
| --- | --- | --- |
|  | **Metaphase** | **Post-mitosis** |
| **siControl** | 319.6 +/- 53 | 364 +/- 51 |
| **siPOC5** | 265.3 +/- 54 | 399 +/- 47 |

**Figure 6-figure supplement 1-source data 1:** Length of centriole in metaphase and at the end of mitosis in siControl and siPOC5 conditions**.**
