# Supplementary material for: WDR90 is a centriolar microtubule wall protein important for centriole architecture integrity
Source: eLife. 2020 Sep 18;9:e57205. doi: 10.7554/eLife.57205 (PMC7500955; doi:10.7554/eLife.57205)
Supplement: Figure 6—figure supplement 1—source data 2. [file elife-57205-fig6-figsupp1-data2.docx]

| **% of cells** | **Conditions** | |
| --- | --- | --- |
|  | **< 2 POC5 dots** | **≥ 2 POC5 dots** |
| **siControl** | 7.1 +/- 3.5 | 92.9 +/- 3.5 |
| **siWDR90/POC5** | 67 +/- 0.4 | 33 +/- 0.4 |

**Figure 6-figure supplement 1-source data 2:** Percentage of cells with the following number POC5 dots/cell in siControl and siWDR90/POC5 conditions.
