# Supplementary material for: WDR90 is a centriolar microtubule wall protein important for centriole architecture integrity
Source: eLife. 2020 Sep 18;9:e57205. doi: 10.7554/eLife.57205 (PMC7500955; doi:10.7554/eLife.57205)
Supplement: Figure 6—figure supplement 1—source data 3. [file elife-57205-fig6-figsupp1-data3.docx]

| **% of cells** | **Conditions** | |
| --- | --- | --- |
|  | **< 2 WDR90 dots** | **≥ 2 WDR90 dots** |
| **siControl** | 7.5 +/- 4.4 | 92.5 +/- 4.4 |
| **siWDR90/POC5** | 54.5 +/- 4.1 | 45.5 +/- 4.1 |

**Figure 6-figure supplement 1-source data 3:** Percentage of cells with the following number WDR90 dots/cell in siControl and siWDR90/POC5 conditions.
